# Supplementary material for: Predictors of Impaired Reperfusion in ST-Elevation Myocardial Infarction Treated with Primary PCI: Preliminary Results from COMA.NET Project
Source: Diagnostics (Basel). 2026 Jan 2;16(1):149. doi: 10.3390/diagnostics16010149 (PMC12785614; doi:10.3390/diagnostics16010149)
Supplement: Supplementary file 1 [file diagnostics-16-00149-s001.zip › diagnostics-4066394-supplementary.pdf]

Predictors of Impaired Reperfusion in ST-Elevation Myocardial Infarction Treated with Primary PCI: Preliminary Results from COMA.NET Project

Supplementary Material

Supplemental Table S1. Univariable regression for prediction of impaired reperfusion.

Variable	OR	95% CI	P-value
Male sex	0.46	0.20–1.08	0.074
Pain time	1.00	1.002–1.007	<0.001
Pain to drug (per increase in 1 min)	1.00	1.002–1.006	<0.001
Off-duty procedure	1.76	0.72–4.26	0.213
Door-to-ballon time	1.01	0.99–1.03	0.529
Pain to balloon time	1.00	1.002–1.006	<0.001
Anterior MI	3.90	1.62–9.41	0.002
Inferior MI	0.27	0.11–0.66	0.004
Sudden cardiac arrest	1.61	0.31–8.42	0.572
Radial access	0.96	0.15–6.00	0.962
Thrombectomy	1.39	0.62–3.13	0.425
Predilation	6.58	2.27–19.08	0.001
Drug eluting stent	1.14	0.54–2.40	0.732
SYNTAX score I	1.05	1.00–1.11	0.040
ADP test on admission	1.00	0.998–1.000	0.168
ASPI test on admission	1.00	0.998–1.001	0.789
Use of GPIIb/IIIa inhibitors	1.42	0.63–3.20	0.403
History of CAD	1.99	0.73–5.46	0.180
Previous MI	0.61	0.11–3.29	0.561
Previous revascularization	0.29	0.03–2.62	0.273
Killip-Kimball Class	5.31	2.28–12.37	<0.001
Smoking	0.22	0.09–0.55	0.001
Hypertension	1.56	0.67–3.66	0.304
Hyperlipidaemia	0.61	0.24–1.52	0.289

Diabetes	1.36	0.51–3.67	0.539
Atrial fibrillation	2.89	0.87–9.60	0.083
Chronic kidney disease	1.83	0.73–4.60	0.199
Body mass index	1.01	0.92–1.11	0.876

Abbreviations: ADP test, adenosine diphosphate induced platelet reactivity test; ASPI test, arachidonic acid induced platelet reactivity test; BMI, body mass index; CAD, coronary artery disease; CI, confidence interval; MI, myocardial infarction; OR, odds ratio; SYNTAX score I, coronary lesion complexity score

Supplemental Table S2. Collinearity diagnostics of the final multivariable model.

Variable	VIF	SQRT VIF	Tolerance	R-Squared
Male	1.2	1.09	0.8351	0.1649
Pain to balloon	1.16	1.08	0.8609	0.1391
Anterior MI	1.38	1.18	0.7242	0.2758
Predilation	1.12	1.06	0.8958	0.1042
SYNTAX score I	1.27	1.13	0.7862	0.2138
Killip Kimball Class	1.63	1.28	0.6117	0.3883
Smoking	1.26	1.12	0.7932	0.2068
Atrial fibrillation	1.17	1.08	0.8526	0.1474

Mean VIF = 1.27

Abbreviations: MI, myocardial infarction; SQRT VIF, square root of variance inflation factor; SYNTAX score I, coronary lesion complexity score; VIF, variance inflation factor.

Supplemental Table S3. Hosmer–Lemeshow analysis of model's calibration.

Group	Probability	Obs_1	Exp_1	Obs_0	Exp_0	Total
1	0.0088	0	0	10	10	10
2	0.0312	0	0.2	10	9.8	10
3	0.1042	1	0.6	9	9.4	10
4	0.1776	0	1.3	10	8.7	10
5	0.2852	3	1.9	6	7.1	9
6	0.4135	5	3.5	5	6.5	10
7	0.6312	5	5.7	5	4.3	10
8	0.7677	7	7	3	3	10
9	0.9591	8	8.8	2	1.2	10
10	0.9996	9	8.9	0	0.1	9

Abbreviations: Exp, expected number of observations; Obs, observed number of observations; Obs_0/Exp_0, observed number of events coded as 0; Obs_1/Exp_1, observed number of events coded as 1.

Hosmer–Lemeshow chi2(8)=4.69

Prob>chi2=0.7903

Supplemental Table S4. Univariable logistic regression investigating platelet reactivity as a predictor of impaired reperfusion. Patients were divided based on pain to balloon time.

Group	Test	N	OR	95% CI	P-value
Pain to balloon <3h	ADP test on admission	30	1.00	0.994–1.002	0.347
Pain to balloon <3h	ASPI test on admission	30	1.00	0.995–1.004	0.804
Pain to balloon <3h	ADP test on 5th day	26	1.00	0.999–1.009	0.117
Pain to balloon <3h	ASPI test on 5th day	24	1.00	0.994–1.005	0.911
Pain to balloon 3-6h	ADP test on admission	37	1.00	0.998–1.001	0.261
Pain to balloon 3-6h	ASPI test on admission	36	1.00	0.998–1.002	0.801
Pain to balloon 3-6h	ADP test on 5th day	30	1.00	0.999–1.006	0.109
Pain to balloon 3-6h	ASPI test on 5th day	30	1.01	0.998–1.013	0.165
Pain to balloon 6-12h	ADP test on admission	19	1.00	0.999–1.003	0.421
Pain to balloon 6-12h	ASPI test on admission	17	1.00	0.996–1.004	0.949
Pain-to-Balloon 6-12h	ADP test on 5th day	18	1.00	0.996–1.003	0.738
Pain to balloon 6-12h	ASPI test on 5th day	14	1.00	0.987–1.012	0.937
Pain to balloon 12-24h	ADP test on admission	12	1.00	0.996–1.010	0.363
Pain to balloon 12-24h	ASPI test on admission	12	1.00	0.992–1.016	0.522
Pain to balloon 12-24h	ADP test on 5th day	12	0.99	0.984–1.002	0.117
Pain to balloon 12-24h	ASPI test on 5th day	10	0.90	0.66–1.24	0.521

Abbreviations: ADP test, adenosine diphosphate induced platelet aggregation test; ASPI, arachidonic acid–induced platelet aggregation test; CI, confidence interval; h, hours; N, number of patients; OR, odds ratio

Supplemental Figure S1. ROC AUC for the prediction of impaired reperfusion of the multivariable model.

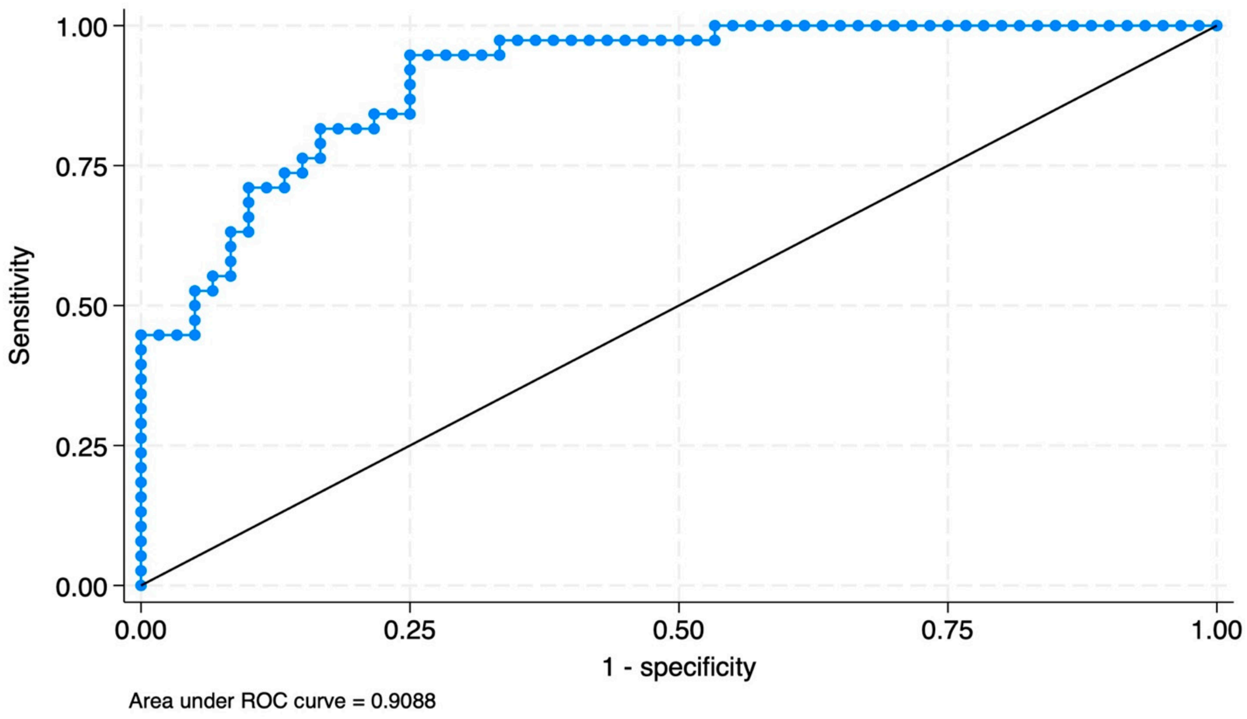

Abbreviations: AUC, area under the curve; ROC, receiver operating characteristic
